# Copper and Anesthesia: Clinical Relevance and Management of Copper Related Disorders

**DOI:** 10.1155/2013/750901

**Published:** 2013-05-13

**Authors:** Adrian Langley, Charles T. Dameron

**Affiliations:** ^1^Department of Anesthesia, Queen Elizabeth II Jubilee Hospital, Locked Bag 2 ARCHERFIELD, Coopers Plains, QLD 4108, Australia; ^2^Department of Chemistry, Saint Francis University, P.O. Box 600, Loretto, PA 15940-0600, USA

## Abstract

Recent research has implicated abnormal copper homeostasis in the underlying pathophysiology of several clinically important disorders, some of which may be encountered by the anesthetist in daily clinical practice. The purpose of this narrative review is to summarize the physiology and pharmacology of copper, the clinical implications of abnormal copper metabolism, and the subsequent influence of altered copper homeostasis on anesthetic management.

## 1. Introduction

Copper is an essential trace element whose absorption, distribution, and elimination are highly regulated. *In vivo*, copper is required for the catalytic activity of several critical enzyme systems. Copper zinc superoxide dismutase (Cu/Zn SOD) acts as a free radical scavenger, cytochrome *c* oxidase functions in energy production, dopamine *β*-hydroxylase catalyzes the conversion of dopamine into noradrenaline, tyrosinase is required for pigment formation, ceruloplasmin is a copper transport protein and anti-oxidant, and lysyl oxidase (LOX) is important for tissue integrity [1]. Copper dependent transcriptions factors may also play an important role in the regulation of cellular proliferation [2].

Clinically defined copper deficiency is rare in western societies; however, altered copper metabolism may influence the conduct and outcome of anesthesia secondary to abnormalities in hemopoietic, cardiovascular, connective tissue, immune and nervous systems. Clinically relevant circumstances predisposing to copper deficiency can include prolonged unsupplemented total parental nutrition (TPN) or enteral nutrition, infants fed with unmodified cow milk based diets, prematurity, gastric bypass and laparoscopic adjustable gastric banding surgery, burns, malabsorption syndromes, and large doses of over the counter vitamins containing zinc and iron [3]. Loss of ceruloplasmin bound copper can also occur in patients undergoing continuous ambulatory peritoneal dialysis for renal failure [4]. Clinical copper toxicity is also rare but is found in genetic overload disorders, occupational exposure, or through ingestion and may also influence anesthetic management through multiorgan failure [5]. 

## 2. Distribution of Total Body Copper Content

Copper is the third most abundant trace element in the body. It is readily absorbed from the diet and is found in foods including legumes, potatoes, grains, shellfish, and beef. Minor sources include tap water, jewellery, intrauterine devices, copper containing pesticides, and cooking utensils. Daily intake is approximately 1.5 mg depending on dietary copper content and regional location [6]. Bioavailability varies from 60 to 70% and inversely correlates with intake. The upper tolerable limit, without the impairment of liver function, is reported to be up to 10 mg per day. Absorption is not influenced by the amount of copper stored in the body (~100–150 mg) [7]. Factors influencing absorption include chemical form of copper, competitive antagonism by other metals (zinc, iron, selenium, cadmium), and malabsorption syndromes resulting from cystic fibrosis, celiac disease, gastrectomy, and jejunoileal bypass surgery. Phytates, dietary cellulose fibre, fructose, and other carbohydrates also reduce copper bioavailability [4, 8]. 

**Figure 1:**
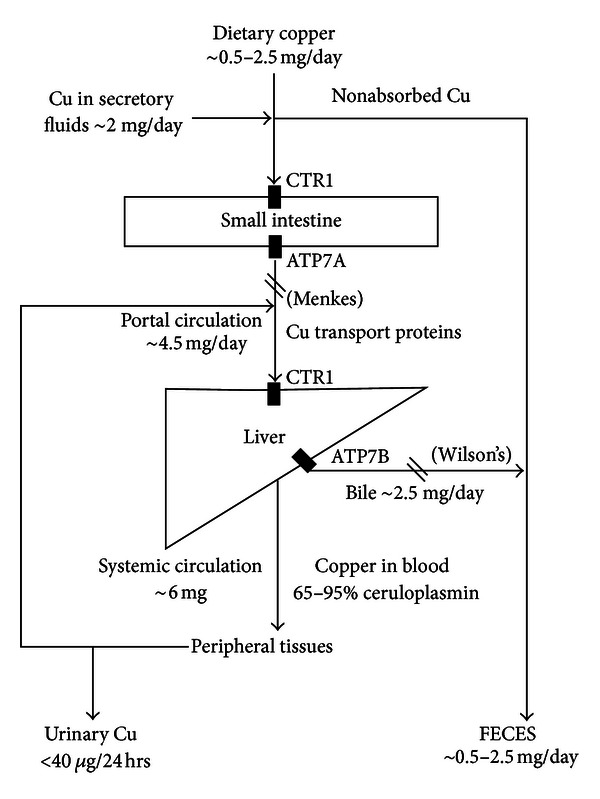
Schematic overview of the major pathways of copper homeostasis. Cu = Copper; CTR1 = Copper Transport Protein 1; ATP7A = Copper transporting ATPase. Mutation is associated with Menkes disease. Encoded by the gene ATP7A; ATP7B = Copper transporting ATPase. Mutation is associated with Wilson's disease. Encoded by the gene ATP7B; Copper transport proteins include fatty acids, albumin, macroglobulin, histidine, and ceruloplasmin. Disruption to copper uptake (Menkes) and excretion (Wilson's) pathways represented by ∖∖.

Tissue copper content varies with gender and age with liver, brain, kidney, and heart containing the highest concentrations. Copper (Cu^1+^) is absorbed primarily across the mucosa of the duodenum and upper small intestine with some evidence for minor amounts of gastric absorption. The copper transport protein Ctr1 is believed to be the major channel through which copper is absorbed with smaller contribution from the divalent metal transporter DMT1 [9]. Transport to peripheral tissues from the liver involves incorporation into ceruloplasmin, which contains 65–95% of plasma bound copper. Excess copper is excreted in the bile (approx 2 mg/day) with very little lost in the urine and 0.5 mg to 2.5 mg per day cleared by defecation (Figure 1) [10]. 

## 3. Mechanism of Copper Toxicity and Detoxification

Copper is a redox active element and its ability to cycle between oxidation states is important in the structural and catalytic roles it plays in various biological systems. Electron transfer is also the mechanism through which copper induces lipid, protein, and DNA damage by the production of reactive oxygen species (ROS) by the Haber-Weiss reaction and Fenton chemistry [11]. 

Copper detoxification can be divided into reduction of metal uptake, enhanced exportation, sequestration, and development of oxidant defense systems. Cation translocating P-type ATPases are involved in the exportation of excessive metals into organelles or out of the cell. Cu/Zn-SOD and Catalase catalyze the conversion of ROS and hydrogen peroxide into less toxic metabolites, respectively. Copper deficiency can lead to a reduction in activity of both enzymes [12]. Glutathione (GSH) is a tripeptide composed of three amino acids: cysteine, glutamic acid, and glycine and acts as an antioxidant and metal chelator. Metallothioneins (MT) are low molecular weight cysteine rich proteins which may act as an antioxidants, metal chelator, or copper chaperones, delivering copper to other intracellular proteins. Copper levels are involved in the regulation of the detoxification response which is regulated at both the transcriptional and enzymatic levels [13]. 

## 4. Menkes and Wilson's Diseases

Menkes disease is a rare X-linked neurodegenerative disorder with an estimated incidence of 1 : 100,00–250,000 births and is characterized by general copper deficiency with death occurring in early childhood, often by three years of age despite treatment with copper histidine supplementation. It is caused by a mutation in the gene encoding P-type ATPase, ATP7A, resulting in defective copper transport across the gastrointestinal tract, placenta, and blood brain barrier [14]. Intracellular copper transport is also abnormal resulting in reduced activity of several copper dependent enzymes. Characteristics of Menkes disease include hypothermia, neuronal degeneration, mental retardation, and abnormalities in hair, skin, and connective tissue [15]. Copper deficiency can be treated orally, intravenously, or subcutaneously. In patients with Menkes disease dosage of copper-histidine used by various authors ranges from 200 to 1000 *μ*g once a day or 2-3 times per week. Therapy is monitored by measuring serum copper, ceruloplasmin, urinary copper excretion, and liver copper content [16]. 

Wilson's disease is an autosomal recessive disorder with an estimated incidence of 1 : 40,000 characterized by hepatic, ophthalmic, and neuropsychiatric symptoms from excess copper accumulation. The molecular pathogenesis is mutation in the P-type ATPase ATP7B gene, which is highly expressed in the liver, kidney, and placenta. ATP7B has two main intracellular roles within the liver; promoting incorporation of copper into apoceruloplasmin and excretion of excess copper into the bile [17]. Unlike many genetic diseases, Wilson's can be effectively treated and early recognition can prevent longer term morbidity from copper induced end organ dysfunction. Therapy includes reducing dietary copper intake, antagonizing its absorption with zinc, or chelation with penicillamine, trientine, or ammonium tetrathiomolybdate. Liver transplantation is indicated where medical therapy is ineffective [18, 19].

## 5. Biomarkers in Diagnosis of Copper Related Diseases

There is no single sensitive and specific test for diagnosing copper related diseases. Plasma copper and ceruloplasmin levels are usually taken as surrogate measurements of body copper storage. Significant changes in free copper may not be detected by measuring plasma copper (normal range 13–22 *μ*mol/L) and ceruloplasmin levels. Estrogen, pregnancy, infections, inflammation, rheumatoid arthritis, and dilated cardiomyopathy reportedly also increase plasma copper levels independent of dietary intake [20]. Erythrocyte Cu/Zn-SOD and platelet or leukocyte cytochrome *c* oxidase may be better indictors of metabolically active copper as their activities are sensitive to copper stores. Other potential biomarkers include assays of copper dependent enzymes including dopamine *β*-hydroxylase activity (DBH), lysl oxidase, and peptidylglycine *α*-amidating monooxygenase (PAM) [21]. There are no laboratory markers that are currently accepted as early markers of copper excess [22]. 

Serum copper and ceruloplasmin levels in Menkes disease are unreliable predictors of copper status in the neonatal period. Reduced activity of dopamine *β*-hydroxylase produces an abnormal plasma neurochemical pattern that may facilitate earlier detection improving treatment outcomes. Although molecular diagnosis is available, this technique is complicated by the diversity of mutations and size of the Menkes gene [23]. In Wilson's disease a spectrum of liver disease may exist from asymptomatic with only biochemical abnormalities to acute liver failure. Abnormal serum aminotransferase levels are common but are relatively nonspecific and do not correlate with the severity of liver disease [24]. Urinary *β*2-microglobulin and N-acetyl-*β*-D-glucosaminidase (NAG) are sensitive markers of renal tubular but are not diagnostic [25]. Ceruloplasmin level is less than 0.2 g/L (normal range 0.2 to 0.5 g/L). Patients may also present with Coombs negative hemolytic anemia and acute renal failure with increased 24-hour urinary copper excretion. Hepatic parenchymal copper content of ≥250 *μ*g/g dry weight, normally < 55 *μ*g/g, remains the best biochemical and diagnostic test. Molecular analysis is potentially diagnostic, but expensive, time consuming and does not necessarily identify all disease causing mutations [26].

## 6. Clinical Importance of Altered Copper Homeostasis in Major Organ Systems Relevant to Anesthesia

### 6.1. Nervous System

Copper is found throughout the brain especially in the basal ganglia, hippocampus, cerebellum, numerous synaptic membranes, and cell bodies of cortical pyramidal and cerebellar granular neurons [27]. It is not known whether copper plays a role in normal synaptic physiology; however, there is evidence suggesting that copper does accumulate in synaptic vesicles, especially glutaminergic neurons and may be co-released with neurotransmitters during normal synaptic events [28]. Postsynaptically copper may interact with receptors and or voltage gated ion channels modulating their activity [29]. Copper acts as a noncompetitive antagonist at N-methyl-D-aspartic acid (NMDA) and Gamma aminobutyric acid A (GABA_A_) receptors. NMDA receptors mediate a wide range of important nervous system functions and are thought to be involved in the development of neuropathic and chronic pain states. *In vitro*, copper has also been shown to promote the downregulation of the NMDA, limiting the entry and accumulation of intracellular calcium, preventing neuronal damage [30]. Copper may also exert an inhibitory effect on neurotransmitter release through inhibition of voltage gated calcium channels by reducing the repetitive firing action potentials of transient and delayed rectifier potassium channels [31]. 

Post translation modification of many peptide hormones and neurotransmitters require copper dependent enzymes for biological activity. Oxytocin, vasopressin, gastrin, corticotrophin-releasing factor (CRF), thyrotropin-releasing hormone (TRH), cholecystokinin (CCK), and calcitonin have significant diminution of bioactivity in the absence of PAM [32]. Abnormal copper transport or aberrant copper-protein interactions may have a role in the development of neurodegenerative diseases. Parkinson's, Huntington's, Amyotrophic Lateral Sclerosis, and Prion diseases all have misfolded proteins that form inclusion bodies involving copper linked processes [33]. 

Copper deficiency associated with myelopathy, known as swayback or enzootic ataxia, is well recognized in ruminant animals. Acquired copper deficiency resulting in “human swayback” is rare but has been reported as a complication of major gastric resection [34]. The neurological features resemble subacute combined degeneration of the spinal cord as occurs in vitamin B12 deficiency. Manifestations include dorsal column dysfunction, progressive numbness, paresthesia, spastic gait, and sensory ataxia. Isolated peripheral neuropathy, central nervous system demyelination, and optic neuritis have also been reported. Somatosensory evoked potentials and nerve conduction studies suggest impaired central conduction and varying degrees of peripheral neuropathy. Early recognition and replacement therapy with oral or parenteral copper may lead to reduced neurological deterioration [35].

### 6.2. Cardiovascular System

Copper deficiency in the cardiovascular system can contribute to hypertension, anemia, coagulation abnormalities, and arteriosclerosis. In animal models dietary deficiency (<1 mg Cu/kg) leads to severe morphological and functional changes. Concentric cardiac enlargement, ventricular aneurysms, mitochondrial disruption, distorted myocytes, and altered proportions of contractile proteins have been documented. Human studies have suggested that marginal copper deficiency may lead to tachycardia, heart block, and premature ventricular beats [36]. 

Patients with Menkes disease may have an increased frequency of congenital heart defects. Prenatal deficiency of lysyl oxidase may prevent the proper formation and stability of the fetal myocardium leading to cardiac abnormalities. Superficial vessels may be tortuous or dilated [37]. In a prospective study of patients with Wilson's disease cardiac copper accumulation was reported to cause left ventricular (LV) parietal thickening with an increased prevalence of concentric LV remodelling and relatively high frequency of benign supraventricular tachycardias and extrasystolic beats [38]. *In vitro* studies suggest that copper chloride (CuCl_2_) may act as an antiarrhythmic, reducing beating rate, action potential amplitude and duration [39].

Copper deficiency leads to morphological changes in the vascular system including altered connective tissue composition and tensile strength leading to aneurysm formation. Vasoactivity of both large and small vessels is altered contributing to derangement in blood pressure homeostasis. Impairment of antioxidant defenses may play a role in development of atherogenesis and ischemic heart disease [36]. 

### 6.3. Skeletal System

Laboratory studies suggest that the neuromuscular blockade by Cu^2+^ is through decreased release of acetylcholine from presynaptic nerve terminals [40]. Myasthenia-like bulbar dysfunction has been reported in a patient diagnosed with Wilson's disease who initially presented with recurrent bleeding which was later followed by swallowing abnormality associated with fatigability [41]. In Menkes disease, muscle biopsies can be nonspecific with changes similar to other myopathies. EMG (Electromyography) findings may vary with the severity of the disease. Early disease EMG results can be normal with more advanced disease demonstrating EMG patterns suggestive of myopathic compromise [42].

### 6.4. Respiratory System

Oxidative stress has been implicated in the pathogenesis of respiratory conditions including asthma, chronic obstructive pulmonary disease, parenchymal lung diseases, and lung malignancies [43, 44]. Cu/Zn SOD, a component of the lungs antioxidant defense system, is highly expressed in type II alveolar cells but poorly expressed in type I Pneumocytes leading to an increased sensitivity to injury and death under conditions of enhanced oxidative stress [45]. Copper deficiency in Menkes disease leads to neonatal emphysema. Several mechanisms have been proposed including impaired cross linking of matrix proteins, as a consequence of reduced lysl oxidase activity (LOX), and derangement of transcriptional mechanisms leading to decreased expression of genes encoding enzymes, growth factors, and matrix proteins [46]. 

Wilson's disease may rarely present as respiratory failure from muscle weakness or hypoxemia from restrictive defects secondary to tense ascites and or pleural effusions, or from ventilation perfusion mismatches associated with liver failure [52]. Hepatopulmonary syndrome (HPS) and portopulmonary hypertension (PPH) are pulmonary vascular disorders which occur in patients with severe liver disease and or portal hypertension. Both conditions are associated with significant morbidity and mortality which may not be improved by liver transplantation [53].

### 6.5. Immune System

Copper deficiency is associated with neutropenia and impaired neutrophil function. Mechanisms may include impaired secretion from the bone marrow, reduced life span, redistribution or early death of progenitor cells, and the presence of antineutrophil antibodies. The ability to generate superoxide anion is also reduced impairing microbicidal activity [54]. There appears to be no effect on circulating levels of complement C3 and C4 in Menkes disease [55]. Macrophage activation triggers increased copper uptake and relocalization of copper transporting ATPase7A to the vesicle, which partially overlaps the phagosomal compartment. Reduced ATPase7A expression attenuates macrophage bactericidal activity and may in part explain the increased susceptibility to respiratory tract infections commonly reported in patients with Menkes disease [56].

The specific acquired immune system consists of Lymphocytes including T (cell mediated) and B (humoral) cells capable of an adaptive targeted response to infection. Copper deficiency reduces anti-body production and cytokine production. Cytokines enable communication between different cells of the immune system. Lower Interleukin 2 (IL-2) levels impairs the proliferative response of splenocytes to mitogens [55].

Chronic long-term copper ingestion modulates the immune response resulting in reduced neutrophil numbers, lymphocyte proliferation, and antigen-specific antibody production [57]. In Wilson's disease there is an increased humoral immune response, with a higher level of IgG and IgM, depressed cell-mediated immunity, and impaired bactericidal activity [58]. Immunosuppression in patients with Wilson's disease and features suggestive autoimmune hepatitis (AIH) may result in initial improvement of liver function; however, in the absence of copper chelation therapy these patients may progress to developing fulminant hepatic failure requiring liver transplantation [59, 60].

### 6.6. Hematopoietic System

Copper deficiency can result in anemia with microcytic or normocytic features, neutropenia, and bone marrow dysplasia. Platelet count may be normal in the presence of pancytopenia. Proposed mechanisms for copper induced anemia include, altered iron metabolism, disordered hemoglobin synthesis, decreased red cell proliferation or increased destruction, and zinc induced malabsorption. Red blood cell survival time is reduced in copper deficiency possibly due to instability of cell membrane, or altered membrane protein and phospholipid altering red cell fragility [61]. 

Coagulation and fibrinolysis are both affected by copper deficiency. Clot formation is delayed; however, thrombi grow more rapidly and lyse more slowly. Copper deficiency impairs endothelial platelet adhesion, enhances platelet aggregation, and delays time to thrombus initiation. Bleeding time is increased and growth phase of the thrombus reduced [36].

Coombs negative hemolytic anemia is an uncommon (10–15%) complication of Wilson's disease. Excess copper released from hepatocyte apoptosis or necrosis is rapidly released into the circulation. In the absence of circulating ceruloplasmin the inorganic copper accumulates within the red blood cells [62]. Copper accumulation may lead to erythrocyte hemolysis by reducing erythrocyte membrane deformability increasing permeability and osmotic fragility, oxidizing hemoglobin, and reducing the activity of several glycolytic enzymes required for adenosine triphosphate (ATP) synthesis [63].

### 6.7. Endocrine System

 Copper induced oxidative stress may contribute to the development of both diabetes and its complications. Advanced glycation end products (AGEs), formed by spontaneous nonenzymatic chemical reactions between carbohydrates and tissue proteins, are one proposed mechanism for the development of diabetic lesions. AGEs accumulate in the vascular, kidney, extra cellular matrix (ECM), and basement membranes. Complications may result from modifications of existing protein structure and function, interaction with AGE specific receptors, and generation of ROS. Accumulation of catalytically active Cu^2+^ binding AGEs in long lived ECM proteins may mediate local oxidative stress leading to tissue damage. Trials involving the copper chelator Triethylenetetramine (TETA) demonstrated efficacy in preventing or reversing diabetic induced changes in both nonclinical models and in patients with type two diabetes mellitus (T2DM) [64, 65]. 

### 6.8. Renal System

Copper is a renal toxin that can induce renal failure by a direct effect on the kidneys or indirectly through hemoglobinuria from intravascular hemolysis and myoglobinuria. Manifestations can include microscopic hematuria, proteinuria, glucosuria, kidney enlargement, uremia, edema, and renal tubular acidosis. Under normal circumstances copper is largely protein bound with little glomerular filtration. Most filtered copper is either reabsorbed into the blood or sequestered into storage vesicles by ATP7A and ATP7B mediated transport, respectively [66]. In Wilson's disease, copper deposits in the epithelium of the proximal and distal convoluted tubules and in the glomerular mesangium. The resulting basement membrane thickening interferes with the resorptive function of the tubule [67]. 

Elevated plasma copper levels have been found in chronic renal failure and patients undergoing both hemo- and chronic ambulatory peritoneal dialysis (CAPD). The mechanism of copper accumulation in renal failure is unknown but may reflect altered hepatic clearance and appears to be independent of the dialysis modality used with similarly elevated copper level in both hemodialysis and CAPD patients [68]. Repeated dialysis exchange may result in copper deficiency in CAPD patients due to loss of ceruloplasmin bound copper across the dialysis membrane. Ceruloplasmin levels are elevated following hemodialysis possibly reflecting its role as an antioxidant and increased oxidative stress in renal failure which is exacerbated by hemodialysis [69]. The literature suggests that hemodialysis is largely ineffective in removing copper from the body; however, plasma exchange may provide a rapid means of inducing a negative copper balance [70].

## 7. Copper and Pregnancy

Copper plays an important role in normal pregnancy and embryogenesis. Serum copper increases early in pregnancy reaching levels at term approximately twice those of nonpregnant women. Lower plasma copper levels have been found in some conditions diagnosed during the first trimester including spontaneous, threatened and missed abortion, and blighted ovum. Serum and placental copper levels and placental lipid peroxides, a marker of oxidative stress, were found to be increased in women with severe preeclampsia [71]. Impaired placental copper trafficking may also play a role in the development of placental insufficiency through reduced expression of ATP7A [72]. 

Excessive copper is teratogenic and has been associated with fetal intrauterine growth restriction and neurological sequelae. Small amounts from intrauterine devices can prevent embryogenesis by blocking implantation and blastocyst development [73]. Patients with untreated Wilson's disease often have spontaneous abortions but there are no consistent reports of fetal abnormalities. Chronic liver disease and resulting hormonal changes may also lead to infertility [74]. Serum copper levels are reportedly higher in women with a history of post partum depression (PPD) compared to nonpregnant women with depression; raising the possibility of using elevated serum copper as a potential marker to identify women with a predisposition to PPD [75].

## 8. Copper Complexes

Copper complexes may have biological activity which has led to research into their use as potential therapeutic agents as antimicrobials, antiviral, anti-inflammatory, antitumour agents, enzyme inhibitors, or chemical nucleases [76]. Copper complexes of nonsteroidal anti-inflammatory agents have shown to be more effective than their parent drugs, demonstrating enhanced anti-inflammatory and antiulcerogenic activity [77]. Complexes of quinolone derivatives have *in vitro* antibacterial activity against strains of *Staphylococcus aureus *[78]. Interaction between Cu^2+^ ions and aminoglycoside antibiotics does not appear to influence their bacterial efficacy but may stimulate the immune system through cytokine production. Immune modulation and generation of ROS may partially explain aminoglycoside induced toxicity [79]. 

Benzodiazepines are used for their sedative-hypnotic properties and may interact with metal ions *in vivo *affecting their therapeutic actions. Copper-Lorazepam complexes are biologically active, have a rapid onset, and duration more prolonged than Lorazepam itself [80]. Intracerebral injections of copper sulfate have been shown to potentiate morphine analgesia in rats. Analgesic effects could be explained by the formation of copper complexes with endogenous opioids which may be capable of both receptor activation and blocking nociceptive stimuli [81].

## 9. Anesthetic Management of Copper Related Disorders

There is no evidence to suggest that acute copper deficiency adversely affects anesthetic management. Little is known about synaptic copper physiology but there is research implicating copper in the modulation of NMDA, AMPA, and GABA receptor functioning [82]. These ligand gated ion channels may represent the molecular site of action of some anesthetic agents and it could be hypothesized that acute changes in neurological copper metabolism could modulate the effect of these agents [83]. Amidated peptides play a critical role in many physiological functions including hypothalamic pituitary axis regulation, thermoregulation, and cardiovascular function. Copper deficiency coupled with genetic defects in PAM could alter the metabolism, distribution, elimination, and potency of anesthetic agents caused by dysregulation in these systems [84]. 

Chronic copper deficiency is more relevant to the anesthetist because of anemia, immunosuppression, bleeding diathesis, and neurological deficits including myelopathy, polyneuropathy, and demyelination [85]. Anemia has been associated with increased postoperative morbidity and mortality, higher incidence of allogenic blood transfusion, and increased risk of postoperative infection [86]. General anesthesia, surgical stress, hypothermia, hyperglycemia, analgesics, and postoperative pain can increase the risk of postoperative sepsis in patients who are already immunosuppressed secondary to copper induced neutropenia [87]. There may be increased risk of neurological injury in patients with existing neuropathy undergoing neuraxial anesthesia or analgesia. Patients may have increased sensitivity to nondepolarizing neuromuscular blockers resulting in prolonged blockade. Suxamethonium could induce life threatening hyperkalemia in patients with polyneuropathy. Neuraxial blockade could result in unpredictable block heights [88–90]. Acute copper deficiency treatment can include intravenous or oral copper sulphate 2 mg/day. Zinc ingestion should also be minimized [91]. 

Anaesthetic considerations for patients with Menkes disease must include seizure and aspiration prophylaxis, intraoperative warming, difficult intravenous access, and hemorrhage secondary to increased vascular fragility (Table 1). Propofol could be considered for induction as there is little evidence for a true epileptogenic effect from its use [92]. Ketamine has been used in infants with neuromuscular disease and hypotonia but is best avoided due to potential for precipitating seizures [93]. Neuraxial blocks are relatively contraindicated. Patients should be intubated to prevent aspiration. Muscle relaxants may not be necessary under deep sevoflurane anesthesia for intubation or the procedure because of muscle hypotonia. Larger doses of vecuronium may be required due to anticonvulsant liver enzyme induction. Cisatracurium, because of its organ independent metabolism, and Rocuronium are possible alternatives [94]. Suxamethonium is best avoided due to potential risk of hyperkalemia [47].

Postoperatively, hypotonia may increase the risk of airway obstruction in the presence of the residual effects of anesthetic agents. Patients have an increased sensitivity to respiratory depressant effects of opioids which should be minimized where possible with multimodal nonopioid analgesics and careful local anesthesia by wound infiltration. Intramuscular and subcutaneous injections should be avoided due to risk of hematoma formation and bleeding. Recovery should be in a well-monitored environment and consideration should be given to admission to a pediatric intensive care unit depending upon the nature, duration, and intraoperative stability of the patient [48].

General, subarachnoid and epidural anesthesia have all been reported in patients with Wilson's disease [49]. General anesthesia may aggravate hepatic function. Volatile anesthetics are cardiac depressants, reducing cardiac output, mean arterial pressure, and liver blood flow. Impaired hepatic function adversely affects the absorption, distribution, metabolism, and elimination of anesthetic drugs. Hypnotic sedatives may have delayed or incomplete metabolism exacerbating postoperative neurological and psychiatric problems. Induction doses of thiopentone in liver disease should be reduced because of a reduction in plasma proteins, increasing the amount of unbound active drug. Duration of action is also prolonged. There is increased sensitivity to sedative and cardiorespiratory depressant effects of propofol; however, clearance is not significantly impaired by liver disease. The metabolism of suxamethonium may be slowed because of reduced pseudocholinesterase. Patients may be more sensitive to neuromuscular relaxants from reduced muscle functioning secondary to the disease, elevated blood copper levels interfering with neuromuscular transmission, or the use of D-Penicillamine. Regional anesthesia may be safe since peripheral nerve transmission is not altered. Neuraxial anesthesia could be considered in the absence of significant coagulopathy or thrombocytopenia [50].

## 10. Conclusion for Clinical Practice

Copper is an essential trace element which plays a fundamental role in the human physiology. Whilst copper deficiency or toxicity may be uncommon in the general population, abnormal homeostasis occurs in several common clinically important disorders which the anesthetist may encounter in their daily practice; especially in the critical ill patient or those who have undergone obesity surgery. It is unlikely that most anesthetists working outside specialty pediatric units will come across Menkes disease; however, this prototypical copper deficiency disorder illustrates the structural and functional importance of copper. Whilst we do not advocate routine perioperative measurement of copper levels, appreciating copper metabolism and disorders of altered copper homeostasis may help the anesthetist anticipate difficulties with peri- and postoperative anesthetic management.

## Figures and Tables

**Table 1:** Summary of the major anesthetic considerations in the management of patients with Menkes and Wilson's diseases. [47–51].

Condition	Anesthetic considerations	Anesthetic management
Menkes disease	Seizures	Preoperatively: Continue anticonvulsant regimen. Check levels. Intraoperatively: Consider alternate routes of administration including intravenous, rectal, and subcutaneous or nasogastric.
	Gastroesophageal reflux	Consider prophylaxis and endotracheal intubation.
	Difficult intravenous cannulation	Use ultrasound for central intravenous access for placement and to identify vascular abnormalities.
	Capillary fragility	Consider group and hold with cross match where clinically indicated.
	Hypothermia	Use warmed intravenous fluids, theatre temperature regulation, forced air warmers, and humidification of inspired gases.
	Neuraxial anesthesia	Relatively contraindicated due to risk of bleeding from vessel fragility.
	Muscle relaxation	May not be necessary under deep volatile anesthesia because of hypotonia. Larger doses of vecuronium may be needed secondary to liver enzyme induction. Suxamethonium may be best avoided due to risk of hyperkalemia.
	Opioid related respiratory depression	Multimodal nonopioid analgesics and careful local anesthesia by wound infiltration. Consider postoperative respiratory monitoring where clinically indicated.
	Post operative analgesia	Risk of bleeding or hematoma formation with intramuscular or subcutaneous routes.

Wilson's disease	Neurological and psychiatric	Delayed metabolism of hypnotic sedative drugs may exacerbate neurological or psychiatric postoperatively.
	Hepatic	Impaired metabolism and elimination of anaesthetic agents and morphine. Reduced mean arterial pressure may further aggravate hepatic function. Propofol clearance not significantly impaired; Reduce Thiopentone dosage.
	Regional or neuraxial anesthesia	Acceptable in absence or significant coagulopathy (INR > 1.4) or thrombocytopenia (platelets < 100,000 mm^−3^).
	Cardiovascular	ECG or echocardiography if coronary artery disease or cardiomyopathy suspected.
	Renal	Fluid and electrolyte abnormalities common. Severe liver dysfunction may result in hepatorenal syndrome which may require dialysis perioperatively.
	Muscular	Avoid or reduce dosage of nondepolarizing neuromuscular blockers (NDMB).
